# Biophysical insights into recombinant Zeocin binding protein: conformational stability and folding dynamics across pH and temperature

**DOI:** 10.3389/fmolb.2026.1748036

**Published:** 2026-01-29

**Authors:** Sara Alharbi, Ajamaluddin Malik, Abdulaziz Alamri, Javed Masood Khan, Mohd. Shahnawaz Khan, Abdullah Alhomida, Tauseef Ahmad

**Affiliations:** 1 Department of Biochemistry, Collage of Science, King Saud University, Riyadh, Saudi Arabia; 2 Department of Food and Nutrition, Facility of Food and Agriculture Science, King Saud University, Riyadh, Saudi Arabia; 3 Department of Medical Education, College of Medicine, King Saud University, Riyadh, Saudi Arabia

**Keywords:** bleomycin, circular dichroism, dynamic multimode spectroscopy, fluorescence, thermal stability, zeocin

## Abstract

Zeocin-binding protein (ZBP) confers resistance to the bleomycin family of antibiotics across a wide variety of prokaryotic and eukaryotic hosts. Zeocin is a bleomycin derivative used in cancer treatment. ZBP’s mechanism involves binding to Zeocin, shielding DNA from damage. In protein folding studies, ZBP, a versatile marker across various organisms, can be used to track protein folding by fusing it to target proteins in prokaryotes and eukaryotes. Despite its significance, ZBP’s biophysical properties are poorly studied. This study characterized the conformational changes, pH stability, solubility, and thermodynamic stability of recombinant ZBP from *Streptoalloteichus hindustanus*. ZBP’s aggregation tendency was assessed across pH ranges, showing notable changes near its isoelectric point (pI) of 4.5. Dynamic multimode spectroscopy analyzed ZBP’s thermodynamic properties at different pH levels, revealing distinct conformations and reversible thermal stress responses. At physiological and alkaline pH levels, ZBP undergoes a single reversible thermal transition between 60 °C and 80 °C with thermal transition midpoints (*Tm*) of 67.4 ± 0.1 °C and 65.4 ± 0.1 °C, respectively. However, at pH 2.0, the *Tm* was reduced to 54.3 ± 0.3 °C. Overall, this study characterized the biophysical stability of ZBP under various pH and thermal conditions, providing essential insights for its optimized application as a selectable marker in molecular biology.

## 1 Introduction

Antibiotic resistance markers have emerged as invaluable tools in molecular biology and protein engineering (Foit et al., 2009; Malik et al., 2014; Tsai et al., 2022). These markers, derived from antibiotic-resistance genes in bacteria, confer resistance to specific antibiotics and are used to select cells carrying the desired genes. In protein engineering studies, they serve as fusion tags. One prominent example of such fusion tag systems is the tripartite fusion system developed by Bardwell and colleagues (Foit et al., 2009). The protein of interest (POI) is fused to an antibiotic resistance marker, such as β-lactamase or kanamycin resistance, allowing selection of cells expressing the fusion protein with the corresponding antibiotic. This strategy has revolutionized protein research by facilitating the manipulation of target proteins. But primarily limited to prokaryotic hosts (Quan et al., 2011; Quan and Bardwell, 2012).

A few antibiotic markers could be used for selection in prokaryotic and eukaryotic hosts, but antibiotics such as Blasticidin S or Hygromycin B are very expensive (Santerre et al., 1984; Izumi et al., 1991). One of the potential examples of such a marker is the Zeocin binding protein (ZBP), which provides resistance against the antibiotic Zeocin (a relatively inexpensive antibiotic) in both prokaryotic and eukaryotic hosts (Bennett et al., 1998). In protein folding studies, ZBP can serve as an invaluable tool. By fusing ZBP with target proteins, researchers can monitor the folding kinetics of the target protein (Bennett et al., 1998; Van Blokland et al., 2011). The resistance to Zeocin conferred by ZBP provides a selective advantage to cells expressing the fusion protein, potentially enabling the isolation and analysis of correctly folded proteins. Furthermore, ZBP’s broad applicability across various host organisms, including bacteria, yeast, mammalian cells, and plant cells, could facilitate comparative studies across different species (Gramany et al., 2016; Takahiro et al., 2021; Zheng et al., 2022). This versatility enables researchers to gain deeper insights into protein folding processes and molecular interactions. Additionally, ZBP can be genetically engineered to optimize its performance as a fusion tag, thereby enhancing its utility in protein engineering.

Zeocin belongs to the bleomycin/phleomycin family of antibiotics obtained from *Streptomyces* verticillus. The bleomycin family of antibiotics members are water-soluble, metal-chelated glycopeptides initially isolated from Actinomycetes (Fujii et al., 1974; Mazzei, 1984; Wang et al., 2021). Zeocin exhibits strong toxicity against a wide range of bacterial, yeast, plant and mammalian cell lines (Drocourt et al., 1990; Zheng et al., 2022). The zeocin formulation contains the active ingredient phleomycin D1, which is a basic copper-chelated glycopeptide. The zeocin antibiotic cleaves explicitly double-stranded DNA, causing cytotoxicity in both prokaryotic and eukaryotic hosts (Dell et al., 1980; Mulsant et al., 1988). The ShBle gene from *Streptoalloteichus hindustanus*, a bleomycin-resistant marker (Drocourt et al., 1990), will be used in this study. ZBP is a 13.6 kDa homodimeric protein that binds stoichiometrically and with high affinity to various antibiotics of the bleomycin family (Gatignol et al., 1988).

This study addressed the critical gap in understanding the biophysical properties of ZBP, a protein with dual significance in cancer therapy and as a versatile marker in protein folding research. The stability of ZBP’s are poorly characterized. The objective of this study was to comprehensively analyze ZBP’s conformational stability, solubility, and pH-dependent thermodynamic properties. Findings reveal ZBP’s pronounced aggregation near its pI of 4.5 and a reversible, single thermal transition at physiological pH, with stability drastically reduced under acidic conditions. This characterization is significant as it provides essential insights for optimizing ZBP’s application in biotechnology.

## 2 Materials and methods

The ZBP codon-optimized sequence was commercially synthesized by GenScript and cloned between NdeI and BamHI restriction sites (www.genscript.com). ZBP was recombinantly expressed in *E. coli* BL21 (DE3)-RIL competent cells (Cat. No. 230240) purchased from Agilent Technologies. SigmaAldrich supplied Benzonase (Cat. No. 70746) and Hen egg white lysozyme (Cat. No. 62971). IPTG (Cat. No. IB0168) and Ampicillin (Cat. No. AB0028) were obtained from Biobasic. The AKTA purification system, Ni–NTA column (Cat. No. 17-5247-01) and LMW markers (Cat. No. 17-0446-01) were acquired from Cytiva, while Invitrogen provided the 4%–12% Nu-PAGE gel (Cat. No. NP0322BOX). All the other reagents utilized in this investigation were of analytical-grade quality. Eppendorf supplied the tabletop chilled centrifuge, Thermomixer, and Innova 44R shaking incubator.

### 2.1 Cloning, expression, and purification of ZBP

The ZBP gene of *Streptoalloteichus hindustanus*, which was modified to match the codon usage of *E. coli*, was inserted into the unique NdeI and BamHI sites on the pET3a vector (Novagen Co.). The clone was named as pET3a-ZBP. Subsequently, it was transformed into chemically competent *E. coli* BL21 (DE3)-RIL cells. The LB and LB agar medium was supplemented with 200 μg/mL ampicillin throughout this study. An isolated individual colony was cultivated overnight in a 20 mL LBamp medium at 37 °C with constant shaking. Subsequently, a 1% (v/v) of overnight culture was inoculated into 1L of LBamp, and the culture was cultivated at 37 °C until it reached the mid-log phase (OD_600nm_ of 0.6). ZBP synthesis was induced by adding 0.1 mM Isopropyl β-D-thiogalactopyranoside (IPTG).

Following a 5-hour induction period, the biomass was harvested using centrifugation at a speed of 4,000 rpm for 30 min at 4 °C. Subsequently, biomass was stored at −80 °C.

To extract soluble ZBP, ∼5 g of wet biomass were suspended in 10 mL of lysis buffer (containing 50 mM Tris at pH 8.0, 2 mg/mL lysozyme and 1 mM EDTA) at 4 °C for 30 min. Subsequently, Nonidet-P 40 (0.5% v/v), glycerol (5% v/v), 300 mM NaCl, and 1 mM PMSF were added and allowed to incubate for 15 min at 4 °C. Afterwards, a solution containing 5 mM magnesium chloride and 1 μL of benzonase was added and left to react for 45 min at 4 °C, until the thickness of the solution was decreased. Subsequently, the lysate was centrifuged at 13,000 rpm for 30 min at 4 °C. The supernatant was then passed through a 0.45 micron filter. The protein was purified in a single-step chromatography with a slight modification of the protocol described in Malik et al. (2016). The imidazole concentration was set to 10 mM in the washing and equilibration buffer. The column had been equilibrated using buffer A (50 mM Tris pH 8.0, 500 mM NaCl, 20% glycerol, 10 mM Imidazole). The soluble protein extract was then passed through a 1 mL Ni-NTA column attached to an AKTA purification system. The column was rinsed with buffer A until the baseline was achieved. Afterward, the column was rinsed with a 5% buffer B (buffer A + 500 mM imidazole) to remove loosely bound proteins, especially metal-binding proteins. The tightly attached protein (his-tagged protein) was subsequently eluted using a gradient ranging from 5% to 50% over a period of 10 min, using buffer B. The fractions containing ZBP were examined using a 4%–12% gradient SDS-PAGE (Invitrogen), and subsequently pooled. The buffer was exchanged, and the protein was concentrated using a centricon. The freshly purified protein was subjected to subsequent biophysical characterization.

### 2.2 ZBP equilibration at various pH values

ZBP (0.2 mg/mL) was equilibrated by overnight incubation at 4 °C in a series of 20 mM buffers covering a pH range of 2.0–12.0: Gly-HCl (pH 2.0–3.0), acetate buffer (pH 4.0–5.0), phosphate buffer (pH 6.0–7.0), Tris (pH 8.0), Gly-NaOH (pH 9.0–10.0), and KCl-NaOH (pH 11.0–12.0) (Malik et al., 2018).

### 2.3 Turbidity measurements of ZBP at different pHs

The impact of various pH levels (ranging from 2.0 to 12.0) on the turbidity of ZBP (at a concentration of 0.2 mg/mL) was evaluated twice by scanning UV-Vis absorption over 250–600 nm. Using a Cary 60 UV-visible spectrophotometer (Agilent Technologies) and a 1.0 cm path-length cuvette, absorbance measurements were recorded for ZBP samples at different pH values. Subsequently, the changes in the non-chromophoric region (at 350 nm) were plotted as a function of pH.

### 2.4 RLS measurements of ZBP at different pHs

Right-angle scattering (RLS) of ZBP (0.2 mg/mL) at different pHs were assessed twice using a Cary 60 UV-visible spectrophotometer in a 1 cm path-length cuvette at room temperature. RLS spectra were obtained by exciting the samples at 350 nm and collecting data over 300–400 nm. The widths of the excitation and emission slits were both set to 2.5 nm.

### 2.5 Intrinsic fluorescence measurement of ZBP at different pHs

The intrinsic tryptophan fluorescence of ZBP was measured at room temperature using a Cary Eclipse Fluorescence Spectrophotometer (Agilent Technologies, California, USA). ZBP samples at a concentration of 0.05 mg/mL, over pH 2-12, were placed in a cuvette with a 10 mm path length. The samples were excited at 295 nm with a slit width of 10 and 5 nm, and the tryptophan fluorescence emission spectra were measured at 5 nm. The data were collected from 300 to 400 nm.

### 2.6 Far-UV-CD measurement of ZBP at different pHs

Far-ultraviolet circular dichroism (CD) spectra of ZBP at various pH values were measured using a ChirascanPlus spectropolarimeter (Applied Photophysics Ltd, UK). The far-UV CD spectra of ZBP were recorded at a concentration of 0.2 mg/mL in a 0.1 cm path-length cuvette at room temperature. Each sample’s spectrum was scanned from 200 to 250 nm with a 1 nm bandwidth, and data were collected at 0.5 s per point. The spectra of ZBP were plotted after subtracting the air baseline and buffer background.

### 2.7 Dynamic multimode spectroscopy of ZBP at different pHs

Dynamic multimode spectroscopy (DMS) was conducted utilizing a ChirascanPlus spectrophotometer. Various pH conditions, including acidic (pH 2.0), neutral (pH 7.0), and alkaline (pH 12.0)were chosen for comprehensive spectroscopic and thermodynamic investigations. ZBP (0.2 mg/mL) was prepared in 50 mM buffer at the pH values, and temperature-induced conformational alterations were examined using an internal temperature sensor in 0.1 cm path length cells. The temperature of ZBP was gradually increased from 20 °C to 94 °C at a rate of 1 °C/min, while far-UV CD spectra were collected over 200–250 nm. The thermal transition data was analyzed using Chirascan Global 3 software supplied by the instrument manufacturer.

### 2.8 Statistical analysis

We conducted a statistical analysis of the data and determined the standard deviation using Microsoft Excel.

## 3 Results

### 3.1 Expression and purification of recombinant ZBP

The ZBP gene sequence from *Streptoalloteichus hindustanus* was optimized to enhance its expression in *E. coli*. During the initial trial, *E. coli* BL21 (DE3) RIL cells carrying the expression plasmid pET3a-ZBP were induced with 1 mM IPTG when the culture reached mid-log phase (OD_600_ 0.6) in LB_amp_ media at 37 °C. After the initiation process, the culture was placed in an incubator and allowed to grow for 5 h at a speed of 150 rpm. In parallel, a negative control experiment was performed, involving uninduced cells containing the plasmid and induced cells lacking the plasmid. Examination of the complete cell lysate using SDS-PAGE demonstrated a notable increase in the expression of ZBP in the culture induced with 1 mM IPTG. Conversely, no expression was found in the negative controls. *E. coli* biomass was grown under conditions optimized for the expression of ZBP. The growth of the induced culture was stopped at an OD_600_ of approximately 2.5 in the large-scale expression experiment. As a result, an approximate yield of 3 g wet biomass per liter of culture was obtained.

**FIGURE 1:**
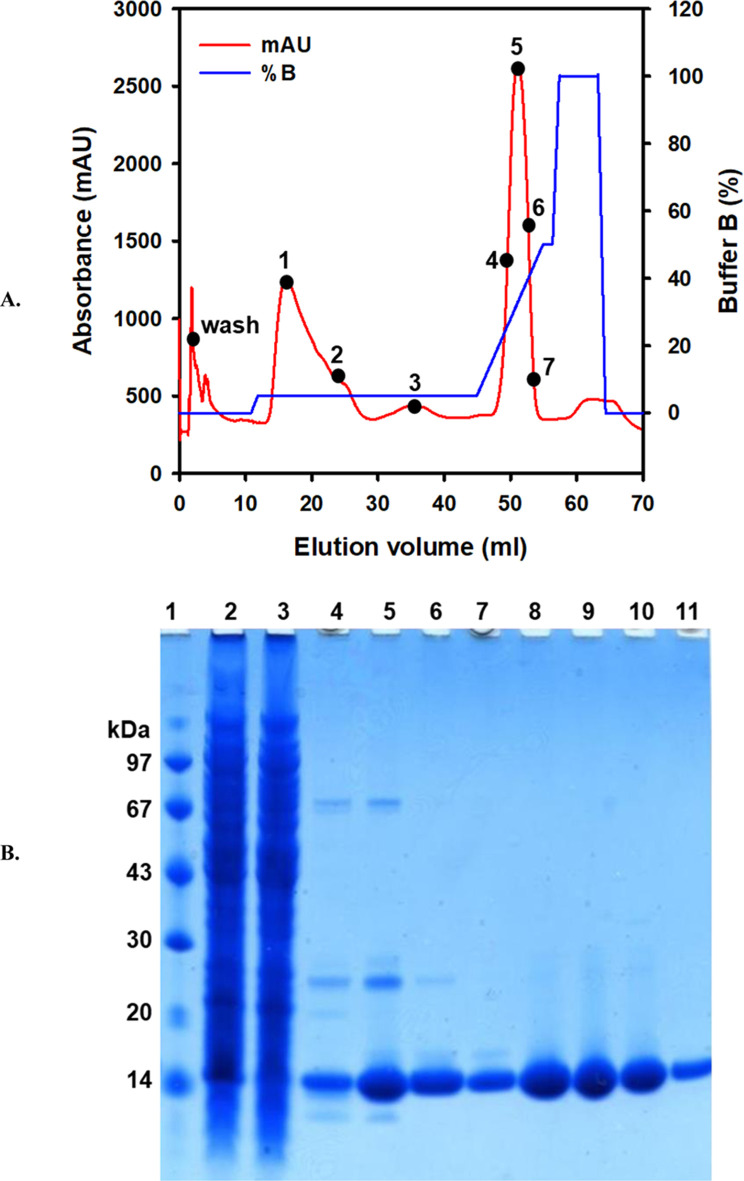
Purification and analysis of ZBP. **(A)** Purification of his-tagged ZBP with Ni-NTA. The soluble protein extract was passed over an equilibrated Ni-NTA column. Loosely bound proteins were eluted using a 0%–5% buffer B gradient. A 5%–50% buffer B linear gradient was used to elute tightly bound protein. The red line represents the protein elution profile, whereas the blue line represents the buffer B gradient. **(B)** The purity of ZBP was analyzed on 4%–12% gradient SDS-PAGE. Lane 1, LMW marker; lane 2, soluble crude extract; lane 3, flow-through; lane 4, wash; lane 5, fraction 1; lane 6, fraction 2; lane 7, fraction 3; lane 8, fraction 4; lane 9, fraction 5; lane 10, fraction 6; and lane 11, fraction 7 (Uncropped image provided in the Supplementary Figure S1).

The soluble protein extract was obtained by treating the biomass with lysis buffer. A potent nuclease (Benzonase) was used to reduce viscosity, followed by centrifugation and filtration through a 0.45 μm syringe filter to clear the crude extract. The ZBP fusion protein contains an N-terminal (His)6-tag. To minimize nonspecific binding, the crude extract was supplemented with 10 mM imidazole and 500 mM NaCl, then passed through an equilibrated HisTrap column (Esteves et al., 2021). The column was initially washed with an equilibration buffer containing 10 mM imidazole to eliminate unbound protein. Subsequently, a 5% elution buffer containing 500 mM imidazole was used to remove weakly bound proteins, such as metal-binding proteins. Finally, 5%–50% gradient was applied to elute strongly bound proteins, such as the his-tagged protein (Figure 1A). SDS-PAGE was used for analyzing the bind, wash, and elute fractions. As shown in Figure 1B (lanes 8–11), the results indicated a high enrichment of ZBP. Consequently, eluted fractions containing relatively pure ZBP were pooled. Following Ni–NTA chromatography, approximately 17 mg of ZBP was obtained from 1L culture.

### 3.2 pH-induced aggregation of ZBP

**FIGURE 2:**
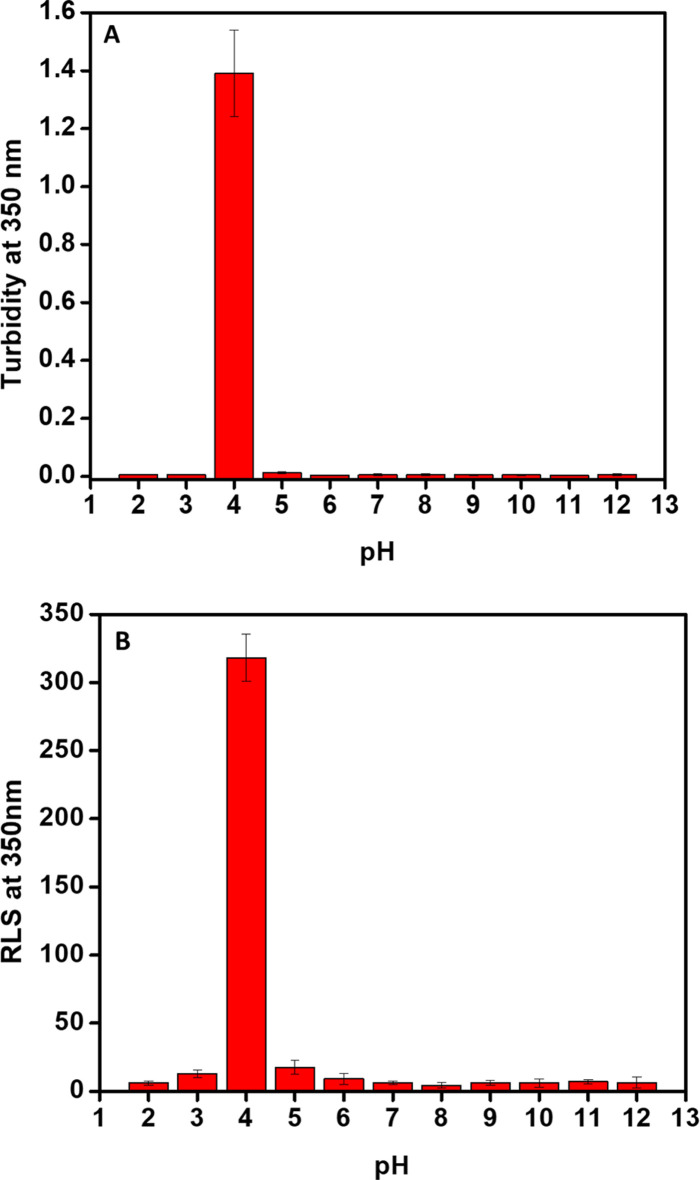
Changes in the aggregation tendency of ZBP to different pH levels. The ZBP solution (200 μg/mL) was equilibrated to pH ranges ranging from 2.0 to 12.0. **(A)** Turbidity data were recorded at various pH values by measuring UV-Vis spectra. The histogram showed the changes in absorbance at 350 nm at different pH conditions. **(B)** RLS was measured at a wavelength of 350 nm, using excitation and emission slits set at a width of 2.5 nm.

The aggregation tendency of ZBP was assessed by measuring its UV-vis absorption in the range of 250–600 nm throughout a pH range of 2.0–12.0. Figure 2 showed the correlation between various pH levels and changes in turbidity. Figure 2A demonstrates a significant rise in the turbidity of ZBP at a pH of 4.0. However, ZBP maintained its solubility across all pH values. Furthermore, the aggregation tendencies of ZBP at various pH levels were assessed using a highly sensitive Right-angle light scattering (RLS) (Khan et al., 2018; Malik et al., 2018). The tendency of ZBP to aggregate in response to alterations was plotted, as shown in Figure 2B. The RLS was measured at a wavelength of 350 nm following excitation at the same wavelength to investigate the aggregation of ZBP under various conditions. The RLS of ZBP at a wavelength of 350 nm was consistent with the baseline for all pH values except for pH 4.0, which indicated that ZBP was insoluble at pH 4.0.

### 3.3 pH-induced changes in the tertiary structure of ZBP

**FIGURE 3:**
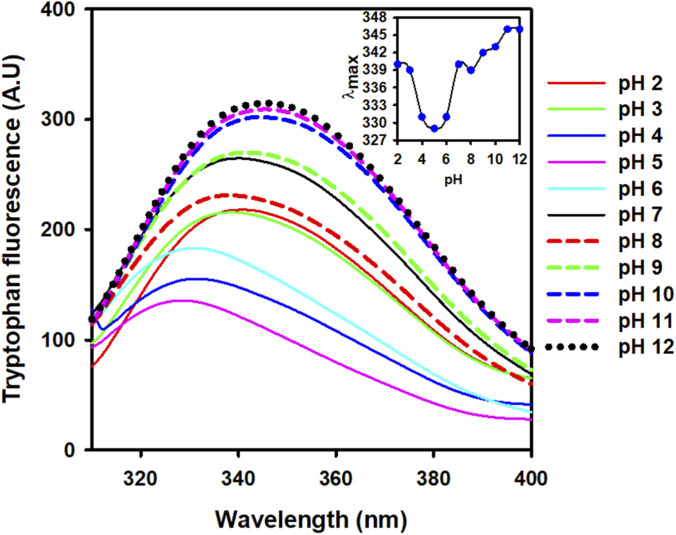
Intrinsic fluorescence spectra of ZBP at different pHs. Tryptophan emission spectra (50 μg/mL) of ZBP were collected at various pH values. The samples were excited at 295 nm, and emission spectra were recorded between 300 and 400 nm. An inset figure displayed the λ_max_ plotted against pH.

The study employed intrinsic fluorescence spectroscopy to examine changes in the tertiary structure of ZBP in response to variations in pH. Measurements of intrinsic fluorescence were advantageous for examining the environment around aromatic residues and yielded insights into even minor alterations in protein tertiary structure (Dos Santos Rodrigues et al., 2023; Alamri et al., 2025). The tryptophan fluorescence spectra of ZBP were showed in Figure 3, spanning a pH range of 2.0–12.0. The ZBP at pH 7.0 showed a peak at 340 nm, suggesting a well-folded structure. The fluorescence emission maximum (λ_max_) of ZBP blue shifted 11 nm as the pH decreased. However, a further decrease in pH led to the restoration of the λ_max_. The native-like λ_max_ value was observed at pH 3.0 and 2.0, as shown in Figure 3 (inset). The blue-shift in the maximum wavelength occurred when the tryptophan residues transitioned from being polar to nonpolar, or when they were buried within the protein’s core or aggregated (Chib et al., 2015). On the other hand, at higher pH (above 7.0), a red shift in λmax was observed, suggesting that the tryptophan residues are exposed to a more polar environment (Duy and Fitter, 2006). The red shift in the wavelength maximum occurred due to the polar (aqueous) microenvironment surrounding tryptophan residues, indicating protein unfolding or loss of protein tertiary structure. The ZBP tertiary structure was shown to undergo changes at pH 11.0 and above, as evidenced by a 6 nm red-shift.

### 3.4 pH-induced changes in the secondary structure of ZBP

**FIGURE 4:**
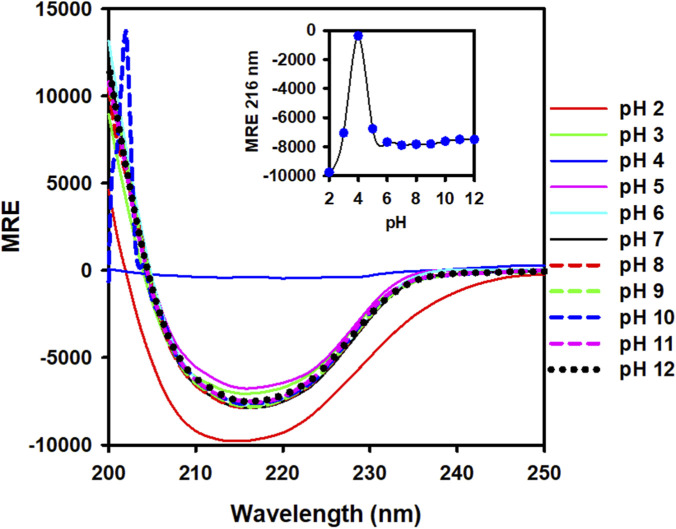
pH-induced changes of the secondary structure of ZBP. The Far-UV CD spectra of ZBP were measured at a 0.2 mg/mL, over a pH range of 2.0–12.0. The inset figure showed changes in the 216 nm ellipticity at different pH levels. The β-sheeted secondary structure was lost when the pH dropped below 6.0, but it quickly recovered a native-like structure when the pH dropped below 4.0. No changes in the secondary conformation of ZBP were detected at pH values above 7.0.

The impact of pH on the secondary structure of ZBP (0.2 mg/mL) was assessed by Far-UV CD measurements in the range of 200–250 nm (Figure 4). The far-UV CD spectra of ZBP at pH 7.0 exhibited negative minima at 216 nm, which is a distinctive attribute of a protein with beta-sheet structure (Greenfield, 2006; Moriyama et al., 2009; Kwan et al., 2022). Negative ellipticity values for ZBP decreased at pH levels below 6.0. The secondary structure was completely lost at a pH of 4.0. The ZBP rapidly reestablished a beta-sheeted conformation when the pH dropped below 4.0, namely at pH 3.0 (Figure 4, inset). The far-UV CD data revealed that the secondary structure of ZBP remained stable at pH 7.0 and above.

### 3.5 pH-induces thermodynamic and spectroscopic studies of ZBP at by dynamic multimode spectroscopy

**FIGURE 5:**
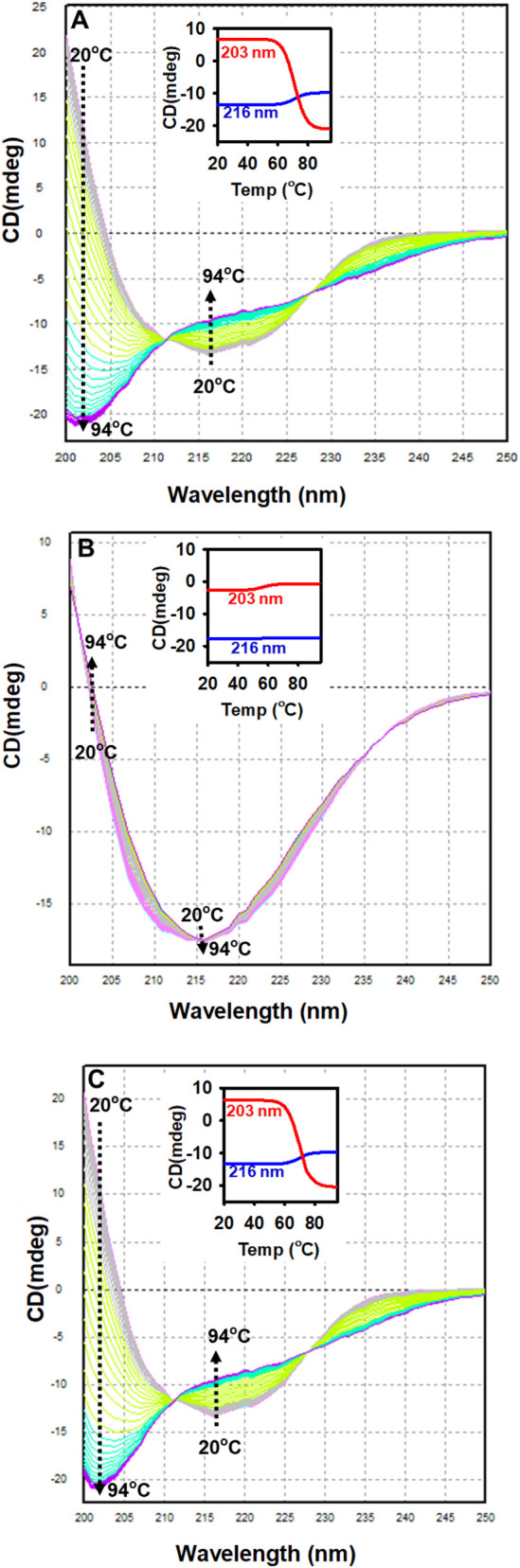
DMS of ZBP at different temperatures and pH values. ZBP (0.2 mg/mL) was heat-stressed at a constant rate (1 °C/min) at pH 7.0 **(A)**, 2.0 **(B)**, and 12.0 **(C)**. Far-UV CD spectra were collected between 200 and 250 nm at 1 °C intervals from 20 °C to 94 °C. In the inset figures, changes at 216 nm are illustrated by a blue line, while a red line with respect to temperature represents those at 203 nm.

The spectroscopic and thermodynamic characteristics of ZBP were analyzed using dynamic multimode spectroscopy (DMS) (John and Weeks, 2000) at three different pHs: acidic (2.0), neutral (7.0), and alkaline (12.0). The ZBP exhibited clearly distinguishable secondary and tertiary conformations at pH levels of 2.0, 7.0, and 12.0. Consequently, these pH values were chosen to assess the thermodynamic and folding properties of ZBP. ZBP samples at a concentration of 0.2 mg/mL were exposed to heat stress from 20 °C to 94 °C at a 1 °C/min rate under similar conditions at pH levels of 2.0, 7.0, and 12.0. The CD spectra in the far-UV range (200–250 nm) were measured at different temperatures. The secondary structural conformation of ZBP was shown in Figures 5A–C, illustrating the variations seen at different temperatures and pH levels. Figure 5A demonstrates that ZBP in its natural state (at pH 7.0 and room temperature) displayed a single minima at 216 nm, indicating a protein with a high content of β-sheet structures. Above 60 °C, the peaks at 216 nm experienced a transition where the β-sheeted conformation changed to a random-coiled structure. A notable observation was made when the temperature exceeded 60 °C: there was a significant rise in the ellipticity minima at 203 nm, with very slight changes at 216 nm. This suggests the emergence of a structure resembling a random coil (Figure 5A inset).

This study also showed that the far-UV CD ellipticity at 216 nm did not change at pH 2.0 (Figure 5B), suggesting that the beta-sheeted structure of the ZBP remained intact even at high temperatures. The far-UV CD spectra of thermally stressed ZBP at pH 12.0 exhibited a resemblance to those of ZBP at pH 7.0 across all temperatures (Figure 5C). The observations indicated the existence of random-coiled structures above 60 °C at pH 7.0 and 12.0. The Far-UV CD spectra of ZBP at pH 7.0 and 12.0 showed similarities. However, the secondary structure of ZBP at pH 2.0 was native-like. Remarkably, the ZBP underwent a transition from its native secondary structure to a random-coiled form at pH 7.0 and 12.0, but remained in a native-like secondary structure at pH 2.0 without undergoing the transition to a random-coiled state. The thermal stress at pH 2.0, 7.0, and 12.0 was reversible, and no aggregation was observed.

**FIGURE 6:**
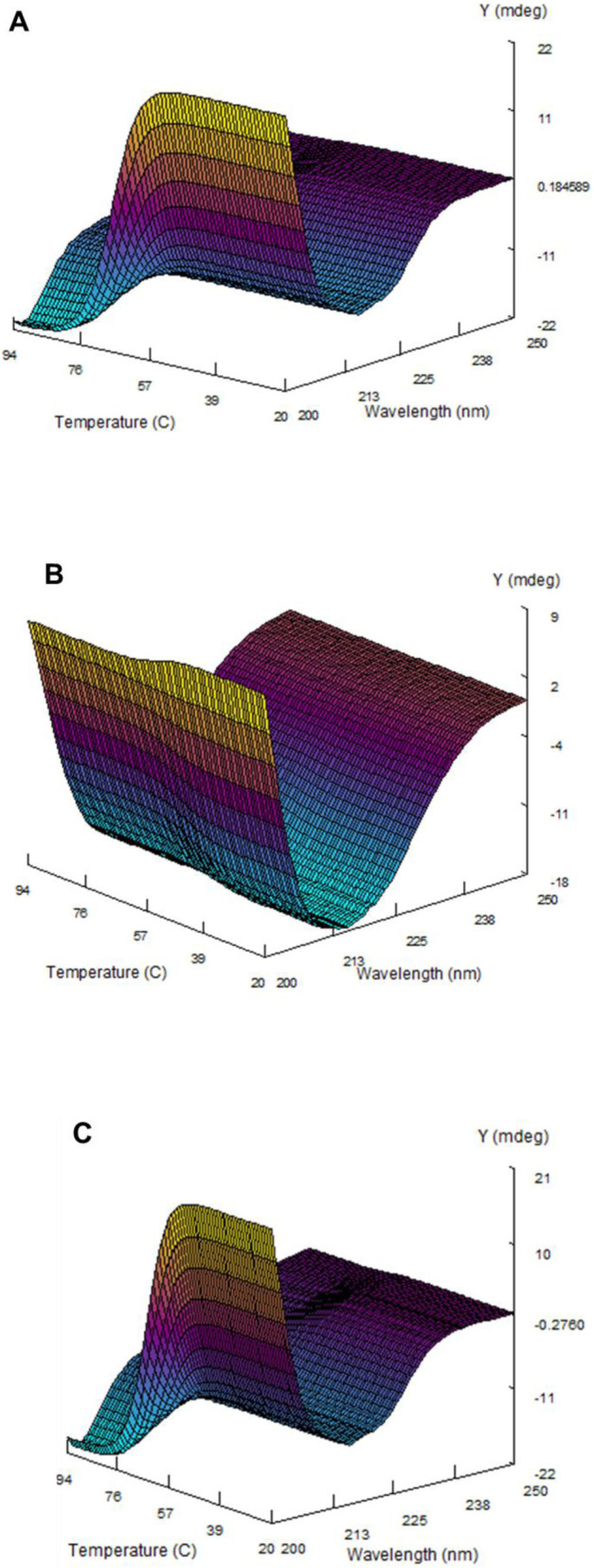
Computed far-ultraviolet circular dichroism (CD), temperature, and wavelength of ZBP at various pH levels. The 3D structure of ZBP at pH 7.0 **(A)**, 2.0 **(B)**, and 12.0 **(C)** was determined using Global 3 software. The structure was computed based on the far-UV CD data obtained while the temperature gradually increased at 1 °C per minute.

The *Tm* values and enthalpy of ZBP were calculated at pH 2.0, 7.0, and 12.0 using the Global 3 software (Table 1). The Global 3 analysis software was used to build a three-dimensional model of the thermal transitions in ZBP at pH 2.0, 7.0, and 12.0 (Figure 6).

**TABLE 1:** Shows the thermal transition midpoints (*Tm*) and Van’t Hoff enthalpies of ZBP at pH 2.0, 7.0, and 12.0, respectively.

pH	Van’t hoff enthalpy (kJ/mol)	Thermal transition midpoints (°C)
2.0	232.9 ± 14.2	54.3 ± 0.3
7.0	267.7 ± 1.6	67.4 ± 0.1
12.0	264.5 ± 1.6	65.4 ± 0.1

## 4 Discussion

Zeocin binding protein, a crucial component in microbial systems, confers resistance to the antibiotic Zeocin (Bennett et al., 1998). Zeocin belongs to the bleomycin family of antibiotics commonly used in cancer treatment because it can induce DNA damage and cell death in cancer cells (Mulsant et al., 1988). In laboratory settings, researchers have used zeocin as a selective agent for cells carrying resistance genes, such as the Zeocin-binding protein, in various prokaryotic and eukaryotic hosts (Zheng et al., 2022). The mechanism of action of the Zeocin binding protein involves its ability to bind zeocin and neutralize its cytotoxic effects. This protein acts as a molecular shield, preventing zeocin from damaging DNA in host cells (Gatignol et al., 1988). Due to its unique properties, ZBP can serve as an invaluable marker in protein folding and engineering studies. One of the key advantages of using ZBP as a marker is its versatility across a wide range of host organisms. Researchers have successfully utilized ZBP in bacterial, yeast, mammalian, and plant cells. This broad applicability enables comparative studies across species, facilitating a deeper understanding of protein folding processes and molecular interactions. Moreover, ZBP can be genetically engineered to be fused with target proteins of interest. By fusing ZBP, the target protein under investigation, researchers can track its folding *in vivo*. The resistance to Zeocin conferred by ZBP provides a selective advantage to cells expressing the fusion protein, allowing for the isolation and analysis of properly folded proteins.

The biophysical properties of ZBP is poorly characterized. Considering the ZBP marker’s wide applicability, researchers in an earlier study engineered the ZBP of *Streptoalloteichus hindustanus* (mesophilic bacterium) to make it a suitable selectable marker for hyperthermophiles (Brouns et al., 2005). The wild-type ZBP was able to confer resistance in *Thermus thermophilus* up to 65 °C growth temperature. The thermal unfolding of purified ZBP by far-UV CD calculated apparent thermal unfolding midpoints (*Tm*) of 70.8 °C ± 0.5 °C (Brouns et al., 2005). This study aimed to characterize the conformational changes, pH stability, solubility, and thermodynamic stability of the ZBP. Initially, the gene sequence of ZBP from *Streptoalloteichus hindustanus* was codon optimized to enhance its expression in *E. coli*. The expression of ZBP was induced in *E. coli* BL21 (DE3) RIL cells using 1 mM IPTG when the culture reached mid-log phase (OD_600_ ∼0.6) at 37 °C. SDS-PAGE analysis of the cell lysate showed a significant increase in ZBP expression in the induced culture compared to negative controls. To purify the ZBP fusion protein, soluble proteins were extracted using a gentle chemical lysis method. The purification protocol was modified to improve the purity of the eluted protein. After applying crude extract on the Ni-NTA column, a wash buffer containing 10 mM imidazole was used to remove unbound and loosely bound proteins. Subsequently, the column was washed with the 5% elution buffer (∼35 mM imidazole) to remove moderately affinity-bound proteins, such as metal binding proteins co-eluted during Ni-NTA purification. Finally, 5%–50% gradient was made to elute the His-tagged ZBP (Spriestersbach et al., 2015). Modifying classical Ni-NTA protocol was promising as SDS-PAGE analysis of eluted protein showed a single band. Thus, ZBP was purified in a single step. Under optimized expression and purification conditions, the yield of purified ZBP varied between 14–19 mg/L culture.

The aggregation tendency of ZBP was assessed across a pH range of 2.0–12.0 using UV-vis absorption and RLS. While turbidity increased significantly at pH 4.0, indicating potential aggregation, no changes in solubility were observed at other pH levels. Intrinsic fluorescence spectroscopy revealed alterations in the tertiary structure of ZBP at different pH values, with tryptophan fluorescence spectra shifting in response to changes in pH, suggesting variations in protein conformation (Ghisaidoobe and Chung, 2014; Dos Santos Rodrigues et al., 2023). Far-UV circular dichroism (CD) spectroscopy was employed to examine the impact of pH on the secondary structure of ZBP (Greenfield, 2006). Negative minima at 216 nm, characteristic of beta-sheet structures, were observed at pH 7.0, with secondary structure lost at pH 4.0 but rapidly restored at pH 3.0. At higher pH levels, the secondary structure remained stable. The calculated isoelectric point (pI) of ZBP is 4.5. The data shown in Figures 1–4, showed that ZBP lost its secondary and tertiary structures near its pI. The loss of a well-folded structure diminishes protein solubility and leads to aggregates.

Proteins near their isoelectric point (pI) lose their secondary and tertiary structures mostly due to alterations in the electrostatic interactions inside the protein (Shaw et al., 2001). Protein conformations are responsive to pH variations due to the ability of charged amino acid residues like lysine, arginine, histidine, aspartic acid, and glutamic acid to either gain or lose protons, which alters the protein’s overall charge distribution (Dharmayanti et al., 2021). Close to the isoelectric point (pI), the protein has a net charge approaching zero, leading to reduced electrostatic repulsion among protein molecules. Consequently, the proteins are more prone to interact via hydrophobic interactions, van der Waals forces, and hydrogen bonding. This results in the formation of protein aggregates (Zhou and Pang, 2018).

Dynamic multimode spectroscopy (DMS) was utilized to analyze the thermodynamic properties of ZBP at acidic (pH 2.0), neutral (pH 7.0), and alkaline (pH 12.0) conditions. This approach identifies changes in the secondary structure of proteins throughout the whole temperature range (Malik et al., 2015; Khan et al., 2016). Figure 6 and Table 1 display the spectroscopic and thermodynamic data acquired by DMS. ZBP exhibited distinct secondary and tertiary conformations at each pH level. The beta-sheeted secondary structure transitioned to a random-coiled form at pH 7.0 and 12.0, while remaining native-like at pH 2.0. The thermal stress experienced at all pH levels was reversible, with no aggregation detected. The thermal denaturation temperatures (*Tm*) and enthalpy values of ZBP were calculated at pH 2.0, 7.0, and 12.0, revealing distinct thermodynamic profiles at each pH. Overall, the study provided comprehensive insights into the structure, stability, and folding properties of ZBP, validating its suitability as a marker for *in vivo* protein folding studies in various host organisms.

At pH 7.0 and 12.0, ZBP existed as a protein with a dominant beta-sheeted structure (Figures 6A,C). However, when subjected to temperatures above 60 °C at these pH levels, the wavelength minima were shifted towards 203 nm. A similar transition was observed in other studies of alpha-crystallin at pH 7.5 (Farnsworth et al., 1997; Malik et al., 2022). Significant conformational changes in ZBP were evident around pH 4.0 (Figure 4). As the pH decreased below 6.0, a decrease in ellipticity at 217 nm was noted when the pH shifted from 6.0 to 4.0. Further pH reduction led to the formation of a native-like beta-sheeted structure in ZBP. Interestingly, at pH 2.0, temperature had no apparent effect on the conformation of the native-like secondary structure of ZBP. A similar observation was also seen in alpha-crystallin at pH 1.0 (Malik et al., 2022).

At physiological and alkaline pH levels, ZBP underwent a single thermal transition occurring between 60 °C and 80 °C. Following heat stress, it remained unclear whether ZBP was fully, partially, or denatured. However, the thermal unfolding process was completely reversible. In this investigation, the midpoint of the thermal transition (*T*_*m*_) was measured to be 67.4 °C ± 0.1 °C at pH 7.0 and 60.9 °C ± 0.1 °C at pH 12.0. The enthalpy of denaturation at these pH levels was calculated to be 267.7 ± 1.6 and 264.5 ± 1.6 kJ/mol, respectively. Due to limited data on ZBP biophysical studies, we were unable to corroborate our findings with prior data. The study provides valuable biophysical insights but has some limitations. As the work was conducted entirely *in vitro* using purified protein, the findings may not fully reflect the protein’s behavior within a living cell. The complex cellular environment, including molecular chaperones, macromolecular crowding, and interacting partners, can significantly influence ZBP’s folding, stability, and aggregation. While the study validates ZBP as a candidate for folding studies, its actual performance and relevance as a marker in specific *in vivo* systems remain to be experimentally confirmed.

## 5 Conclusion

In protein folding research, ZBP serves as a valuable marker due to its versatility across different host organisms, including bacteria, yeast, mammalian cells, and plant cells. By genetically engineering ZBP to be fused with target proteins, researchers can monitor folding kinetics and conformational changes, aiding in the isolation and analysis of properly folded proteins. Despite its importance, the biophysical properties of ZBP remain poorly characterized. Future research should focus on comprehensively characterizing ZBP’s biophysical properties to enhance understanding of its mechanism of action and its potential applications across diverse biological contexts. Additionally, exploring the molecular interactions of ZBP with Zeocin and other ligands could provide insights into its binding mechanism and aid in the design of novel therapeutic agents.

## Data Availability

The original contributions presented in the study are included in the article/Supplementary Material, further inquiries can be directed to the corresponding author.
