# Supplementary figures and images for: Biophysical insights into recombinant Zeocin binding protein: conformational stability and folding dynamics across pH and temperature
Source: Front Mol Biosci. 2026 Jan 29;13:1748036. doi: 10.3389/fmolb.2026.1748036 (PMC12893952; doi:10.3389/fmolb.2026.1748036)

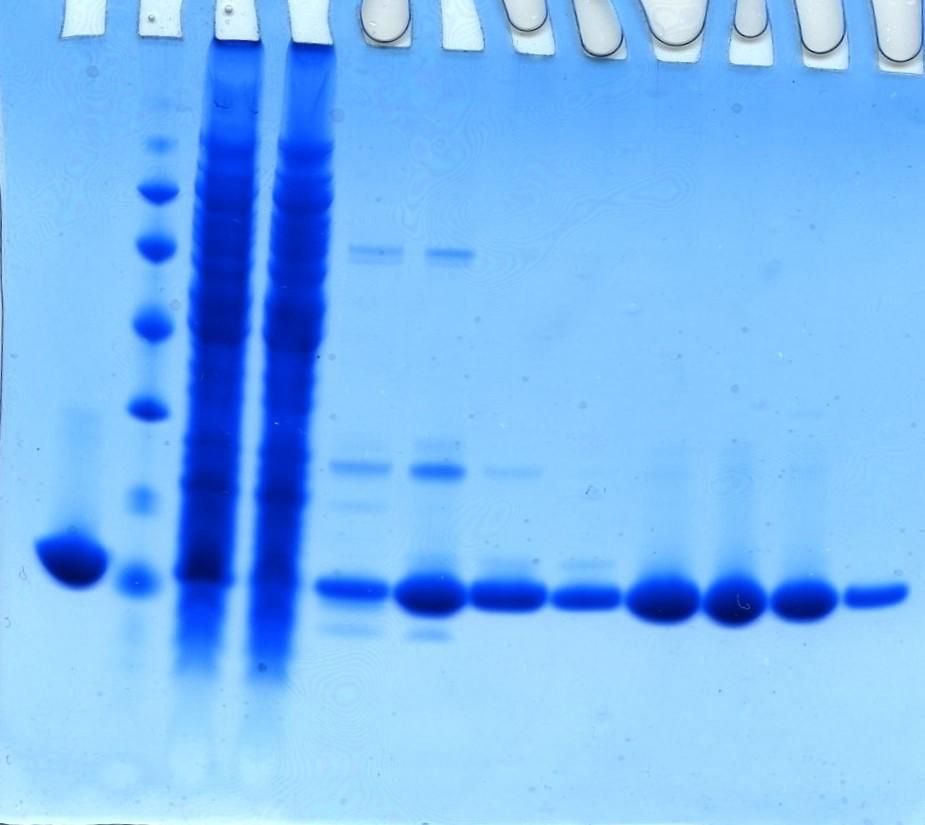

Supplement: Supplementary file 1 [file Image1.jpeg]
